# Exploration Novel Therapeutic Targets for Periodontitis via Stress Granules Biomarkers

**DOI:** 10.1016/j.identj.2025.109390

**Published:** 2026-01-22

**Authors:** Yu Wang, Xie Yang, Bowei Shi, Bowen Zhu, Hai Zhuang, Jialu Chen

**Affiliations:** a Department of Stomatology, Nanjing Drum Tower Hospital, Affiliated Hospital of Medical School, Nanjing University, Nanjing, Jiangsu, China; b Department of Stomatology, the First Affiliated Hospital of Nanjing Medical University, Nanjing, Jiangsu, China

**Keywords:** Periodontitis, Stress granules, Biomarkers, Bioinformatics, Machine learning

## Abstract

**Background:**

Periodontitis (PD) is associated with stress granules (SGs), which are involved in cellular stress responses. Identifying biomarkers related to SGs in PD is key to grasping its pathogenesis and devising novel therapeutic approaches.

**Methods:**

Microarray datasets GSE10334 and GSE106090 were downloaded from public databases, and experimental verification was conducted. Differentially expressed genes (DEGs) were determined via differential expression analysis and intersected with SGs-related genes (SGs-RGs). Machine learning methods and gene expression validation were used to further refine the biomarkers. Subsequently, a nomogram was constructed for disease prediction, and its accuracy was evaluated. Enrichment analysis was performed to investigate signalling pathways, and immune cell infiltration was analysed. Moreover, a transcription factor (TF) regulatory network and a disease-biomarker-drug interaction network were constructed.

**Results:**

A total of 1618 DEGs, 95 candidate genes, and 4 biomarkers *(PECAM1, IL18, EGFR*, and *CCL5*) were identified. *PECAM1* and *CCL5* showed significant overexpression, while *IL18* and *EGFR* showed significant underexpression in PD patients. The nomogram based on these biomarkers showed high predictive accuracy. Enrichment analysis revealed that the biomarkers were primarily accumulated within translation pathways, like ribosome and rRNA processing. Besides, 14 differentially infiltrated immune cell types were identified, with significant positive correlations between *IL18*/*EGFR* and memory B cells, *PECAM1*/*CCL5* and plasma cells. Notably, TFs such as CEBPB, ELF1, and STAT3 were identified as important regulatory factors for the biomarkers. Potential drugs for the biomarkers included mycophenolate, afatinib, and fluticasone, and the biomarkers were associated with diseases such as gingivitis, diabetes, and cardiovascular diseases.

**Conclusions:**

This study identified four SGs-related biomarkers in PD and proposed potential therapeutic targets, providing preliminary insights into its pathogenesis that warrant further experimental validation at the protein and functional levels.

## Introduction

Periodontitis (PD) is a chronic inflammatory disease initiated by bacterial infection and characterised by the progressive destruction of periodontal tissues.^1^ It is characterised by deepening of periodontal pockets, alveolar bone resorption, tooth mobility, and malocclusion, ultimately representing the leading cause of tooth loss in adults.^2^ Emerging evidence has linked severe PD to systemic comorbidities, including cardiovascular diseases, diabetes mellitus, and adverse pregnancy outcomes.^3^, ^4^, ^5^ In terms of pathogenesis, besides the involvement of pathogenic bacteria, the host's immune response serves as a critical mediator in the progression of periodontal tissue destruction.^6^

Stress granules (SGs) are non-membrane-bound structures that primarily assemble through the aggregation of proteins and RNAs under diverse stress conditions, including physical stresses (hypoxia, heat shock, osmotic pressure changes), oxidative stress, and pathogenic challenges (viral infections). The assembly of SGs serves as a highly conserved cellular strategy to mitigate stress-related damage and support cell survival.^7^ Disruptions in SGs assembly and disassembly processes are linked to neurodegenerative diseases, dysregulated antiviral responses, and carcinogenesis, underscoring the critical physiological importance of SGs.^8^ Recent research has demonstrated that while the majority of physiological SGs disassemble following stress resolution, a considerable proportion are cleared via autophagy during prolonged stress exposure.^9^^,^^10^ Given that SGs play crucial roles in innate immune regulation, particularly in response to viral infections, most SG research has focused on virology. Although research into the relationship between bacterial infections and SGs is still in its infancy, bacteria are closely associated with SGs formation and modulate intracellular p-eIF2ɑ levels.^11^ Furthermore, similar to cellular stress responses and viral infections, SGs are interconnected with cellular inflammatory responses. For example, recent studies have shown that SGs mitigate macrophage-mediated inflammatory damage and further regulate cell survival and pyroptosis by inhibiting inflammasome activation.^12^ Research has shown that the inflammatory response in periodontitis is associated with increased local and systemic oxidative stress, as well as a diminished antioxidant capacity.^13^ Recently, the role of regulating mitochondrial abnormalities and reactive oxygen species (ROS) production has gained increasing recognition in the pathogenesis of PD.^14^ In summary, we propose that SGs may play important roles in periodontitis pathogenesis and progression; however, research in this area remains limited, and the precise underlying mechanisms require further elucidation.

The study integrated transcriptomic data and bioinformatics methods to identify biomarkers related to PD and SGs, explored their regulatory mechanisms and signalling pathways, and analysed their correlation with immune infiltration. The findings aimed to assess biomarker potential, reveal molecular mechanisms, and provide theoretical insights for PD therapy development.

## Materials and methods

### Data collection

The GSE10334 and GSE106090 microarray datasets for PD were downloaded from the Gene Expression Omnibus (GEO) database (https://www.ncbi.nlm.nih.gov/geo/) and used as training and validation sets, respectively. Specifically, for the GSE10334 dataset on the GPL570 platform, 154 gingival tissue samples were selected after removing duplicate samples, including 90 PD patient samples and 64 healthy samples. The inclusion criteria were: aged ≥13 years, non-smoking, non-pregnant, no history of systemic periodontal treatment, no use of systemic antibiotics/anti-inflammatory drugs in the previous 6 months, having ≥24 teeth with ≥4 teeth showing radiological bone resorption. Additionally, a total of 12 gingival tissue samples, including 6 PD patients and 6 healthy human samples, were selected from the GSE106090 dataset on the GPL21827 platform. The inclusion criteria were: aged ≥18 years, non-smokers, no known systemic diseases, excluding those who had used antibiotics/anti-inflammatory drugs 3 months prior to surgery. The raw signal values of both datasets were first subjected to outlier processing (removing negative values and zero values), followed by log₂ transformation, and the core normalisation method was quantile normalisation, implemented via the normalizeQuantiles function in the limma package. Furthermore, to explore the potential role of SGs in PD, 844 SGs-related genes (SGs-RGs) were obtained from the literature.^15^

### Differential expression analysis

To identify differentially expressed genes (DEGs) between the PD group and healthy group, differential expression analysis of the expression matrix of the training set GSE10334 was performed using the ‘limma’ package. ^16^ Adjusted *P*-values were calculated via the Benjamini-Hochberg method, with adjusted *P*-value < .05 and |log_2_ fold change (FC)| > 0.50. Subsequently, to visualise the distribution of DEGs, a volcano plot was created via the ‘ggplot2’ package (v 3.4.1).^17^ Additionally, to further illustrate the expression levels of DEGs, a heatmap of the top 10 upregulated and top 10 downregulated genes, ranked by |log_2_FC| from high to low, was plotted using the ‘ComplexHeatmap’ package (v 2.14.0).^18^

### Identification and function analyses of candidate genes

To identify candidate genes associated with SGs in PD, the ‘ggvenn’ package (v 0.1.9) was utilised to find the intersection of DEGs and SGs-RGs and to plot a Venn diagram.^19^ The intersecting genes were defined as candidate genes. Subsequently, to investigate functions of candidate genes and the biological processes they were involved in, Gene Ontology (GO), covering biological process (BP), cellular component (CC), and molecular function (MF), as well as Kyoto Encyclopedia of Genes and Genomes (KEGG) enrichment analyses (*P* < .05) were performed on the candidate genes using the ‘clusterProfiler’ package (v 4.2.2).^20^ The top 5 pathways (ranked by p.adj from lowest to highest) for each category were visualised using the ‘ggplot2’ package (v 3.4.1). Then, to analyse the protein-level interactions of candidate genes, a protein-protein interaction (PPI) analysis was conducted via the Search Tool for the Retrieval of Interacting Genes/Proteins (STRING) (https://string-db.org/), where an interaction score exceeded 0.4. Subsequently, the weights of individual genes within the PPI network were calculated using maximal clique centrality (MCC), maximum neighbourhood component (MNC), degree, edge percolated component (EPC), closeness, and radiality algorithms in Cytoscape software (v 3.8.2) with the CytoHubba plugin.^21^ The top 20 genes with the highest weights for each of the six algorithms were visualised, and their intersection was taken as the key candidate genes for subsequent research.

### Machine learning and expression validation

To further screen candidate biomarkers, in the training dataset GSE10334, least absolute shrinkage and selection operator (LASSO) regression analysis was performed on key candidate genes using the ‘glmnet’ package (v 4.1-8). Ten-fold cross-validation was employed during the analysis. Based on the minimum lambda value in the model, LASSO feature genes were selected according to the criterion of non-zero coefficients in the regression model. Extreme gradient boosting (XGB) analysis was conducted on key candidate genes using the ‘xgboost’ package (v 2.1.1.1) with 100 fixed iterations. Genes with an average gain greater than 0 were filtered out and identified as XGBoost feature genes.^22^^,^^23^ The intersection of genes obtained from two machine learning methods was identified using the ‘ggvenn’ package (v 0.1.9) as the candidate biomarkers for subsequent analysis. To further determine the expression of the candidate biomarkers in PD patient samples and healthy samples, the Wilcoxon rank-sum test was applied to evaluate expression differences of the candidate biomarkers between two groups of samples based on the training dataset GSE10334 and the validation dataset GSE106090 (*P* < .05), and the ‘ggplot2’ package (v 3.4.1) was utilised for visualisation. Finally, those genes that were significantly differentially expressed (*P* < .05) and had consistent expression trends in both datasets of PD patients and healthy samples were defined as biomarkers.

### Construction and evaluation of a nomogram

To explore the predictive value of biomarkers for PD disease, a nomogram was constructed for the biomarkers based on the PD patient samples and healthy samples from the training set GSE10334, using the ‘rms’ package (v 6.8-1).^24^ To assess the accuracy of the nomogram, the ‘pROC’ (v 1.18.0) and ‘regplot’ (v 1.1) packages were used to perform receiver operating characteristic (ROC) analysis [area under the curve (AUC) > 0.7] as well as calibration curve analysis.^25^^,^^26^

### Biomarker distribution and correlation analyses

To identify the specific locations of biomarkers on chromosomes, the ‘RCircos’ package (v 1.2.2) was used to analyse and determine their positioning within the chromosomes.^27^ Subsequently, to ascertain the cellular localisation of the biomarkers, FASTA sequences of biomarkers were obtained from the National Centre for Biotechnology Information (NCBI) website (https://www.ncbi.nlm.nih.gov/), which were then input into the mRNA Locater database (http://bio-bigdata.cn/mRNALocater/) to obtain predictive scores. Following this, based on all samples from the training set GSE10334, the ‘psych’ package (v 2.1.6) was employed to perform a Spearman correlation analysis among the biomarkers (|correlation coefficients (cor)| > 0.30 and *P* < .05).^28^

### Gene set enrichment analysis

To explore signalling pathways involved in the progression of PD by the biomarkers, GSEA was carried out based on PD patient samples and healthy samples from the training set GSE10334. First, background gene set c2.cp.all.v7.0.symbols.gmt was sourced from the Molecular Signatures Database (MSigDB, https://www.gsea-msigdb.org/gsea/msigdb). Subsequently, the relationship between the biomarkers and all other genes was calculated using the ‘psych’ package (v 2.1.6) (|cor| > 0.30 and *P* < .05). Then, all genes were ranked according to the correlation coefficients in descending order to form the related gene set. Finally, GSEA was performed with the ‘clusterProfiler’ package (v 4.2.2) (| normalised enrichment score (NES)| > 1, *P* < .05).

### Immune infiltration analysis

To explore differences in the immune microenvironment between PD patient and healthy groups, based on two groups of samples in the training set GSE10334, relative distribution proportions of 22 different immune cells in each sample were calculated using CIBERSORT algorithm (v 0.1.0) (samples with *P* < .05 were excluded), and the infiltration levels of immune cells were visualised using the ‘ggplot2’ package (v 3.4.1).^29^^,^^30^ Subsequently, to further identify immune cells with significant differences in infiltration between the PD patient group and the healthy group, the Wilcoxon test was utilised (*P* < .05), and boxplots were drawn for visualisation using the ‘ggplot2’ package (v 3.4.1). To analyse correlations between differentially infiltrated immune cell types as well as between biomarkers and differentially infiltrated immune cell types, based on all samples in the training set GSE10334, Spearman correlation analysis was performed via the ‘psych’ package (v 2.1.6) (|cor| > 0.30 and *P* < .05).

### Regulatory network analyses

To investigate the transcription factors (TFs) regulatory mechanisms of biomarkers, the TFs that could regulate the biomarkers were predicted using the online tools Human Transcription Factor Target Database (hTFtarget) (https://guolab.wchscu.cn/hTFtarget/#) and ChIP-X Enrichment Analysis 3 (ChEA3) (https://maayanlab.cloud/chea3/). Intersection of prediction results from the two tools was obtained using the ‘ggvenn’ package (v 0.1.9), and then the TF-mRNA regulatory network was built with Cytoscape software (v 3.8.2). Subsequently, potential drugs related to the biomarkers were forecasted using Drug-Gene Interaction Database (DGIdb) (https://dgidb.org/), and the associations between the biomarkers and PD-related diseases were further analysed using the Comparative Toxicogenomics Database (CTD) (https://ctdbase.org/). Finally, the disease-biomarker-drug interaction network was visualised using Cytoscape software (v 3.8.2).

### Clinical samples collection and quantitative real-time polymerase chain reaction (qRT-PCR) analysis

Gingival samples were collected from 6 healthy individuals and 6 PD patients, respectively. The demographic and clinical characteristics of participants are listed in Supplementary Table 1. The samples were immediately stored in liquid nitrogen. The classification criteria for periodontitis were set in the 2017 World Workshop on the Classification of Periodontal and Peri-Implant Diseases and Conditions.^2^ Exclusion criteria encompassed scaling and root planning within the previous 6 months, the utilisation of specific medications (such as immunosuppressive drugs, antibiotics, or nonsteroidal anti-inflammatory drugs), systemic conditions that could affect the progression of periodontitis, pregnancy or lactation, smoking, having fewer than 20 teeth, and inability to provide consent.^31^ This study was conducted at the First Affiliated Hospital of Nanjing Medical University, received approval from the Ethical Committee of the First Affiliated Hospital of Nanjing Medical University (2024-NT-09), and complied with the 1964 Declaration of Helsinki and subsequent amendments and ethics standards.

After sufficient grinding with liquid nitrogen, total RNA was extracted from the gingiva by using RNAiso Plus (Takara). Subsequently, 5 x PrimeScript RT Master Mix (Takara) was utilised for reverse transcription, and a TB Green PCR Core Kit (TaKaRa) was employed for PCR amplification. The reaction and detection were implemented on a CFX96TM system (Bio-Rad). The relative mRNA expression levels of each target gene were calculated by using the 2 ^-ΔΔCT^ method. The primer sequences of genes used in the study are shown in Table. Subsequently, an independent samples t-test was conducted to evaluate the expression levels of the biomarkers in diseased and healthy samples by using SPSS 25 software (IBM Corp.). *P* < .05 was considered statistically significant.

**Table:** Primer sequences information used in this study.

Gene	Forward primer sequence (5′-3′)	Reverse primer sequence (5′-3′)
*GAPDH*	GGAAGCTTGTCATCAATGGAAATC	TGATGACCCTTTTGGCTCCC
*EGFR*	AGCTACGGGGTGACTGTTTG	GTCTCGGGCCATTTTGGAGA
*IL18*	AAAGATAGCCAGCCTAGAGGTATG	TTATCATGTCCTGGGACACTTCTC
*CCL5*	CCCTCGCTGTCATCCTCATTG	TCGGGTGACAAAGACGACTGC
*PECAM1*	CACCAAGATAGCCTCAAAGTCGG	ACCTCAAACTGGGCATCATAAGAA

### Statistical analysis

All statistical analyses were carried out with R statistical software (v 4.2.2). Differences between the two groups were compared using the Wilcoxon rank-sum test or Student's *t* test, as appropriate (*P* < .05).

## Results

### Function exploration of 95 candidate genes

**Fig. 1:**
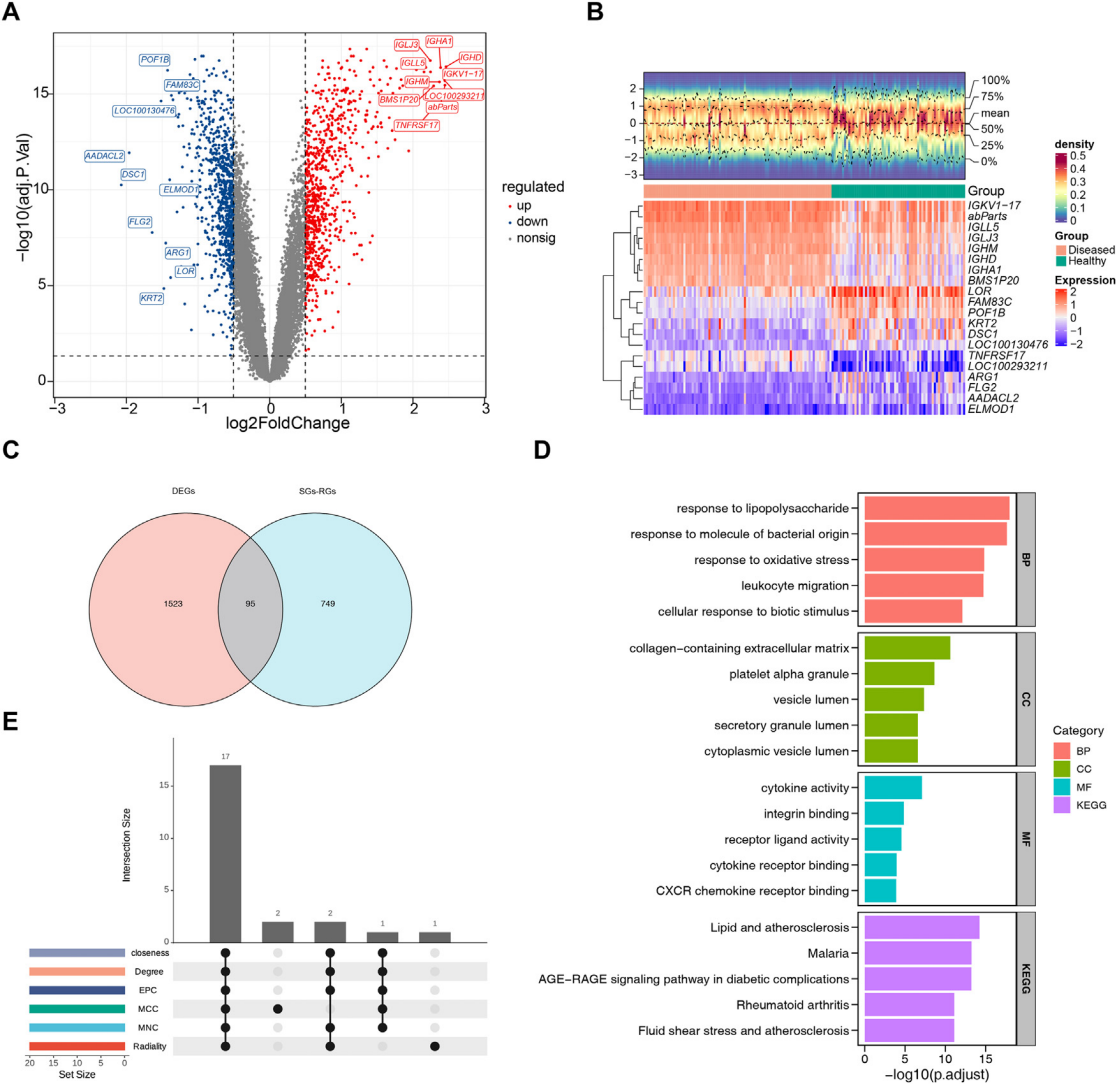
Identification of candidate genes. (A) Volcano plot of differentially expressed genes between the PD group and the control group. Upregulated genes are marked in red; downregulated genes are marked in blue. (B) Heatmap of differentially expressed genes. Red indicates highly expressed genes, and blue indicates lowly expressed genes. (C) Venn diagram of candidate genes of PD and SGs. (D) GO pathway and KEGG pathway analysis of the top 5 pathways. (E) Identification of Key candidate genes in the PPI network across six algorithms.

This study ultimately identified 1618 DEGs. Compared with the healthy samples, there were 776 upregulated genes and 842 downregulated genes in PD (Figure 1A, Supplementary Table 2), and an expression heatmap of the top 10 upregulated and top 10 downregulated DEGs was displayed (Figure 1B). Following this, 95 candidate genes (Figure 1C) were obtained from the intersection of 1618 DEGs and 844 SGs-RGs.

Subsequently, significant enrichment of 1134 GO terms and 100 KEGG pathways (Supplementary Table 3) was discovered among the candidate genes (*P* < .05) (Figure 1D). Among these, there were 1067 BP terms, such as response to lipopolysaccharide, cellular response to biotic stimulus; 38 CC terms, such as platelet alpha granule, vesicle lumen; and 29 MF terms, such as cytokine activity, CXCR chemokine receptor binding. KEGG enrichment included pathways like malaria, AGE-RAGE signalling pathway in diabetic complications, rheumatoid arthritis, fluid shear stress, and atherosclerosis. These results indicated that the candidate genes were involved in key roles such as inflammatory responses, immune responses, and oxidative stress in PD.

Moreover, a PPI network was built to depict the interactions among the candidate genes at the protein level. Importantly, some candidate genes, such as *IL6, IL1B, MMP9*, and *CXCL8*, were found to play key roles in the PPI network across 6 algorithms, and 17 key candidate genes were screened (Figure 1E).

### Identification of biomarkers

**Fig. 2:**
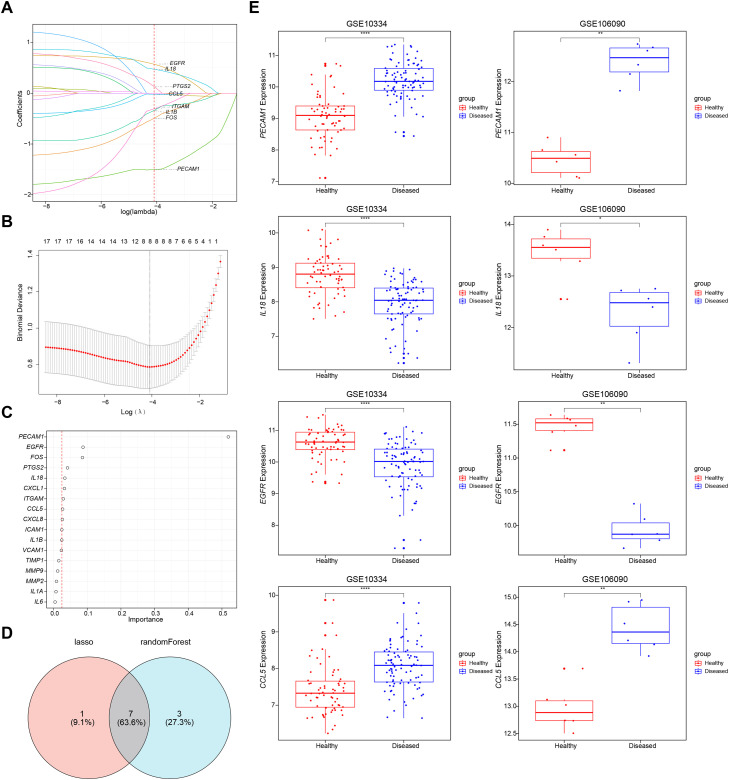
Identification of biomarkers via machine learning algorithms. (A-C) Identification of diagnostic biomarkers by LASSO analysis and XGB analysis. (D) Venn diagram for the two algorithms. (E) Validation chart of biomarker expression levels.

Based on the 17 key candidate genes, the LASSO analysis identified 8 feature genes (Lambda min = −4.09) (Figure 2A and B), and the XGB analysis obtained the top 10 feature genes ranked by importance (Figure 2C). The intersection of these analyses yielded 7 candidate biomarkers (*FOS, EGFR, PECAM1, IL18, CCL5, ITGAM, PTGS2*) (Figure 2D). In the expression validation, *PECAM1, IL18, EGFR*, and *CCL5* showed significant differences and consistent expression trends in both datasets (*P* < .05), and they were defined as biomarkers (Figure 2E). Specifically, *PECAM1* and *CCL5 s*howed significant overexpression, whereas *IL18* and *EGFR* showed significant underexpression in PD patients, providing a solid theoretical foundation for the treatment of PD.

### Diagnostic model for biomarkers

**Fig. 3:**
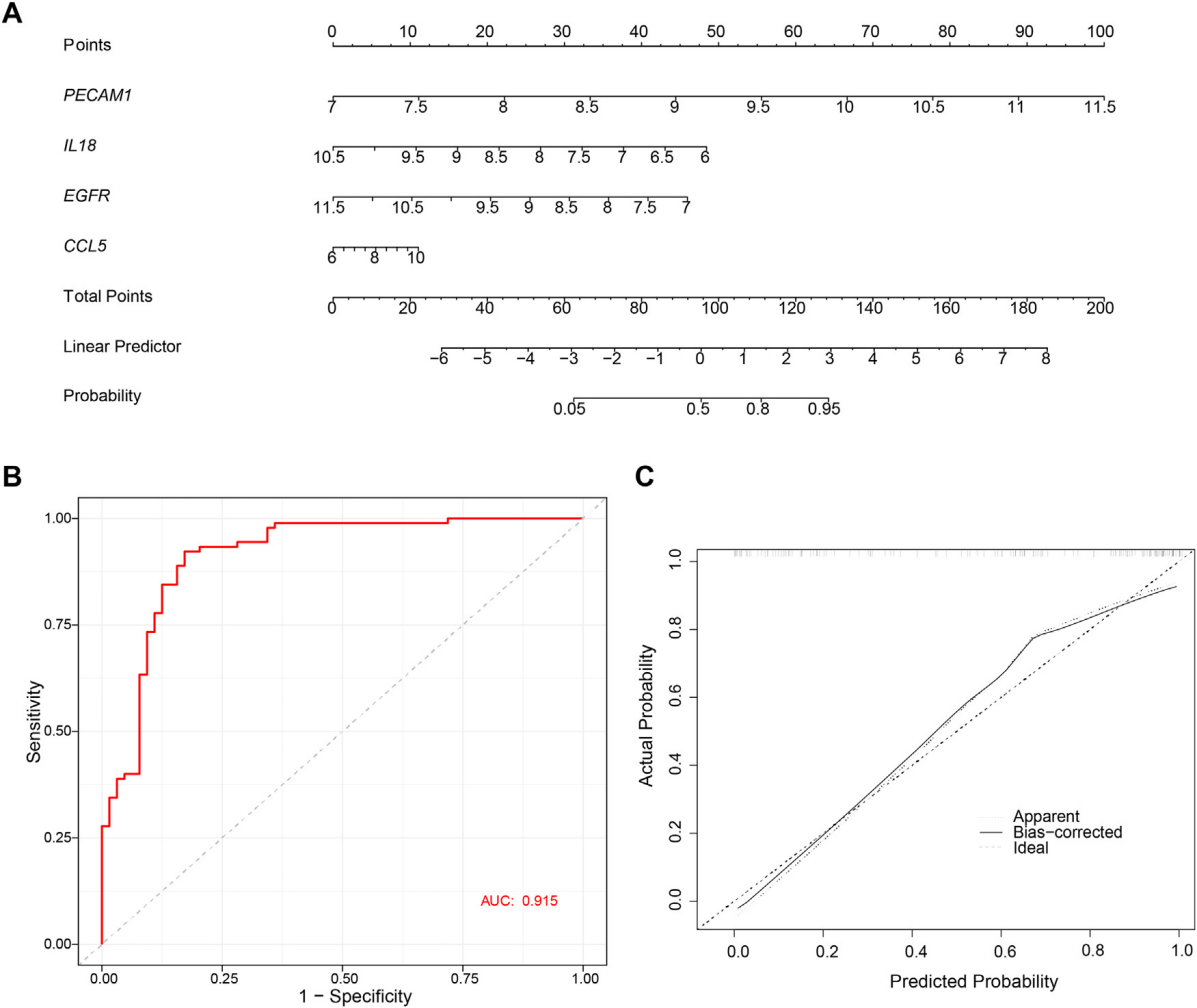
Evaluation of diagnostic biomarkers. (A) Construction of the nomogram model based on the four biomarkers. (B) ROC curve of nomogram. (C) Calibration curve of the biomarker nomogram.

To evaluate the risk of PD, a nomogram was constructed based on the four biomarkers *PECAM1, IL18, EGFR*, and *CCL5* (Figure 3A). The AUC value of the ROC curve was 0.915, which preliminarily indicated that the nomogram had high accuracy (Figure 3B), and the slope of the calibration curve was close to 1, indicating that the predicted results of the model had good consistency with the actual clinical outcomes (Figure 3C). This helped improve the diagnostic accuracy and therapeutic outcomes for PD.

### Chromosomal, subcellular localisation, and correlation analyses of biomarkers

**Fig. 4:**
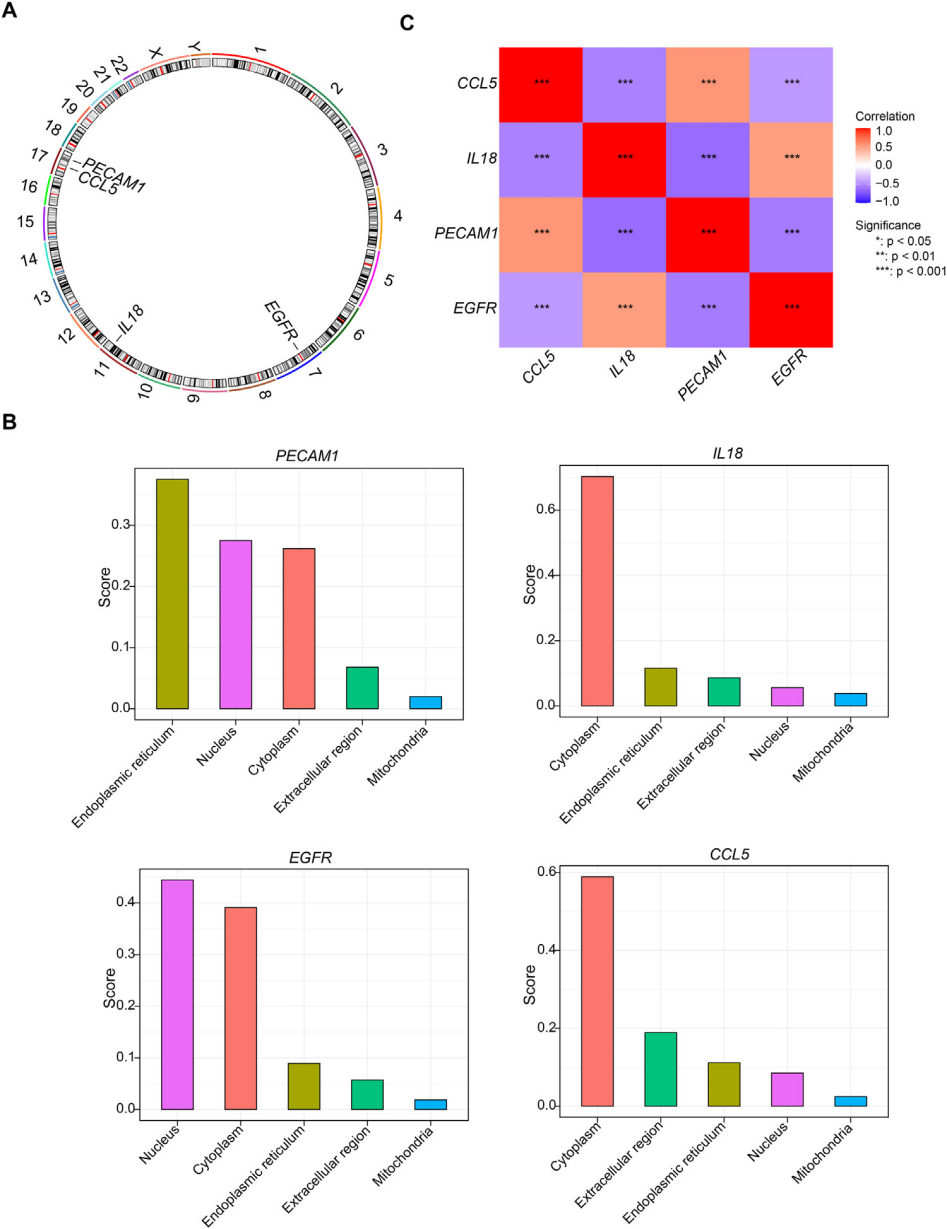
Chromosomal, subcellular localisation, and correlation analyses of biomarkers. (A) Biological marker chromosome localisation. (B) Subcellular localisation of four biomarkers. (C) Heatmap of biomarker expression correlation.

The chromosomal localisation results indicated that *PECAM1* and *CCL5* were both located on chromosome 17, while the other genes were located on different chromosomes, with *IL18* on chromosome 11 and *EGFR* on chromosome 7 (Figure 4A). Subsequently, the subcellular localisation results showed that PECAM1 was primarily found in the endoplasmic reticulum, IL-18 and CCL5 were mainly in the cytoplasm, and EGFR was predominantly in both the nucleus and cytoplasm (Figure 4B). All biomarkers exhibited significant correlations in gene expression levels (|cor| > 0.30, *P* < .001) (Figure 4C). Specifically, *IL18* and *CCL5, PECAM1* and *IL18*, as well as *EGFR* and *CCL5, PECAM1*, were significantly negatively correlated (cor < −0.30, *P* < .001). On the other hand, *PECAM1* and *CCL5*, as well as *EGFR* and *IL18*, were markedly positively correlated (cor > 0.30, *P* < .001).

### Enrichment analysis of biomarkers via GSEA

**Fig. 5:**
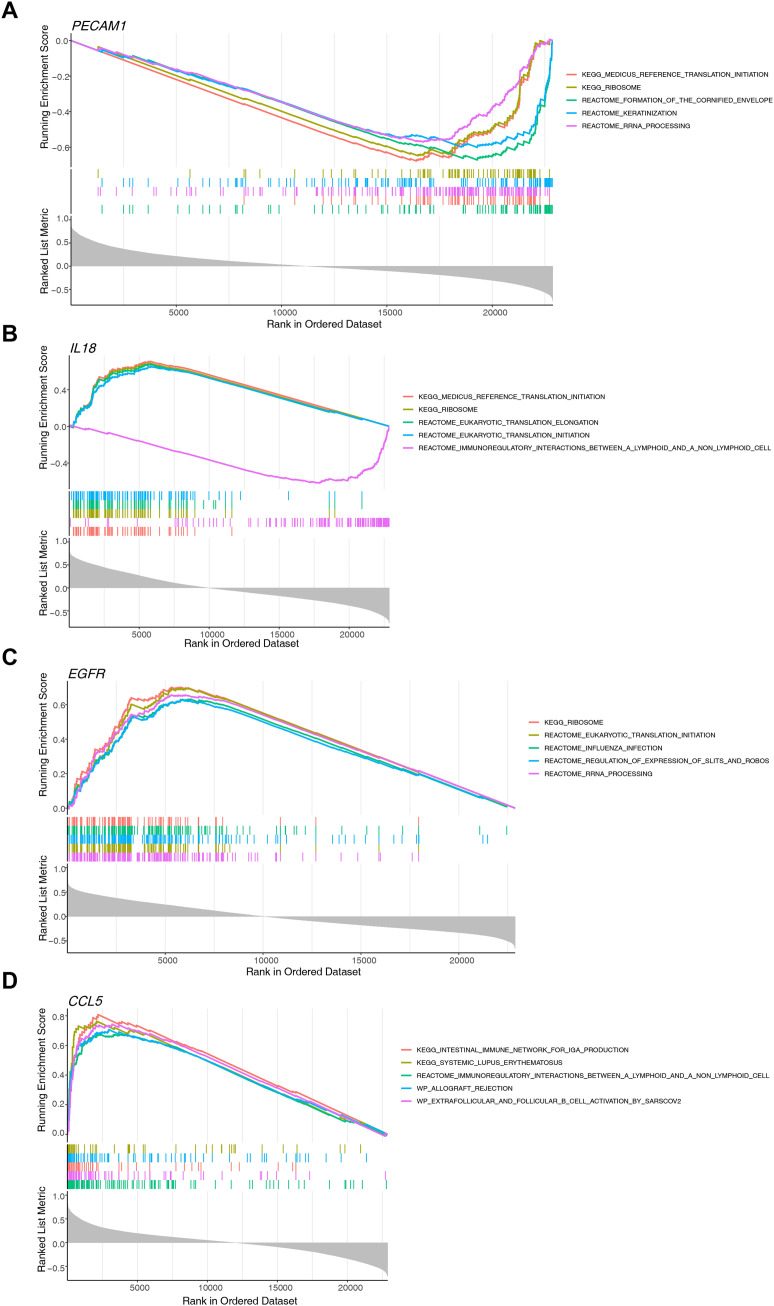
GSEA showed the top 5 KEGG signalling pathways of (A) *PECAM1*, (B) *IL18*, (C) *EGFR*, and (D)*CCL5*.

GSEA outcomes revealed that *PECAM1* was significantly featured in 777 pathways (*P* < .05), such as reference translation initiation, ribosome, formation of the cornified envelope, keratinisation, and rRNA processing (Figure 5A and Supplementary Table 4). *IL18* was significantly enriched in 938 pathways (*P* < .05), such as reference translation initiation, ribosome, eukaryotic translation elongation, and eukaryotic translation initiation (Figure 5B and Supplementary Table 4). *EGFR* was significantly enriched in 1209 pathways (*P* < .05), such as ribosome, influenza infection, and rRNA processing (Figure 5C and Supplementary Table 4). *CCL5* was significantly enriched in 871 pathways (*P* < .05), such as allograft rejection, and extrafollicular and follicular B cell activation by SARS-CoV-2 (Figure 5D and Supplementary Table 4). In summary, the biomarkers were primarily concentrated in translation-related pathways like ribosome, reference translation initiation, and rRNA processing. This suggested that these biological processes might play crucial roles in the PD disease mechanism.

### Immunological feature analysis

**Fig. 6:**
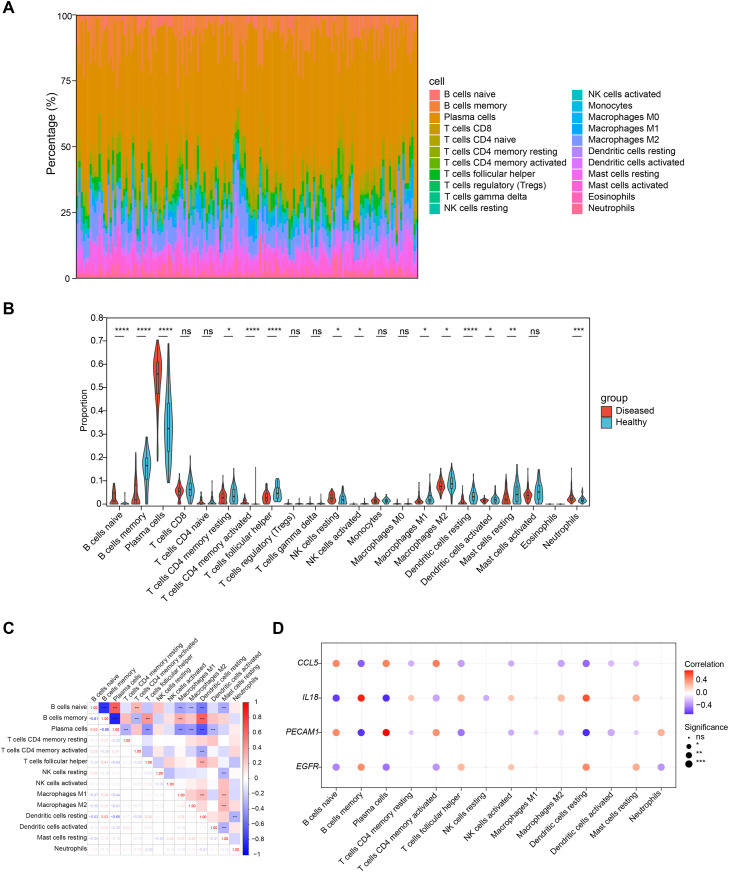
Correlation between biomarkers and immune cell infiltration levels in the PD microenvironment. (A) The infiltration levels of immune cells in PD patients and healthy controls. (B) Immune cell differences between PD patients and healthy controls. (C) Heatmap of correlations among different immune cells. (D)Bubble diagram of biomarker-immune cell correlations.

The infiltration levels of immune cells in PD patient samples and healthy samples are shown in Figure 6A. Fourteen types of immune cells exhibited noticeable infiltration differences between PD patients and healthy groups (Figure 6B) (*P* < .05), such as naive B cells, memory B cells, plasma cells, activated CD4 memory T cells, T follicular helper cells, and resting dendritic cells. Some differentially infiltrated immune cell types were significantly positively correlated, such as between plasma cells and naive B cells (cor = 0.65, *P* < .001), between resting dendritic cells and memory B cells (cor = 0.63, *P* < .001), while some differentially infiltrated immune cell types were significantly negatively correlated, such as between plasma cells and memory B cells (cor = −0.86, *P* < .001) (Figure 6C). All biomarkers were significantly positively or negatively correlated with some differentially infiltrated immune cell types, such as *PECAM1* and plasma cells (cor = 0.72, *P* < .001) or resting dendritic cells (cor = −0.65, *P* < 0.001) (Figure 6D and Supplementary Table 5). The changes in these immune cells might have reflected the complex immune response mechanisms in PD.

### Construction of TFs-mRNAs network and disease-biomarker-drug interaction network

**Fig. 7:**
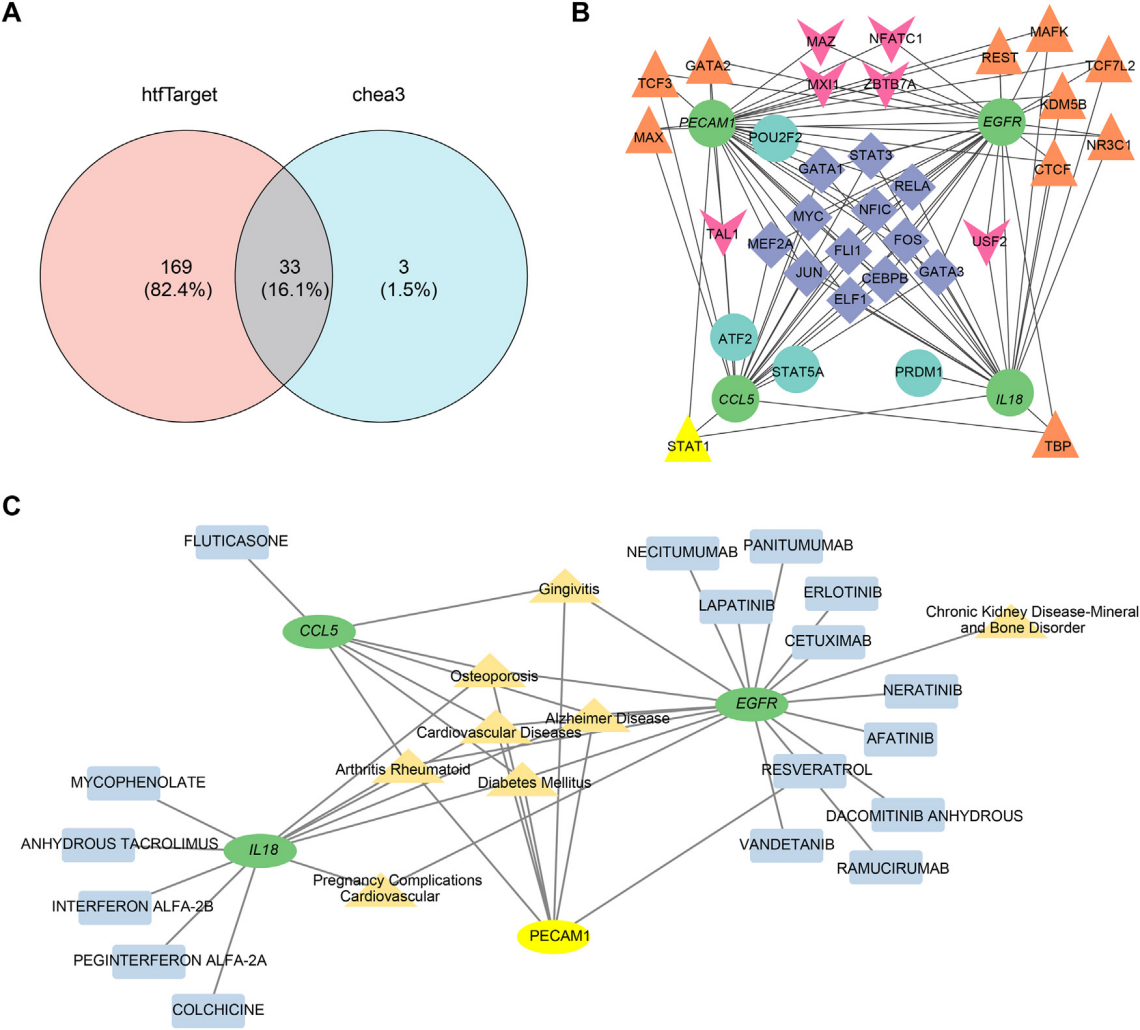
TFs-mRNAs network and disease-biomarker-drug interaction network. (A) Venn diagram showing TFs overlap between hTFtarget and CheA3. (B) Biological markers and TFs network diagram. Green circles represent biomarkers; blue circles indicate TFs targeting one gene; pink arrows show those targeting two genes; orange triangles represent those regulating three genes; and purple rhombuses denote those influencing four genes. (C) Drug interaction with biomarkers in PD-related diseases.

A total of 33 intersecting TFs were obtained from the hTFtarget and ChEA3 websites (Figure 7A). The TFs-mRNAs network was shown in Figure 7B, where TFs such as CEBPB, ELF1, FLI1, STAT3, and GATA1 could target the biomarkers *PECAM1, IL18, EGFR*, and *CCL5* simultaneously. The disease-biomarker-drug interaction network is shown in Figure 7C. For the biomarkers, *PECAM1* had the potential compound resveratrol; *IL18* was associated with the potential drugs mycophenolate, anhydrous tacrolimus, and colchicine; *EGFR* was associated with the potential drug afatinib, necitumumab, and cetuximab; *CCL5* was associated with the potential drug fluticasone. All these biomarkers were associated with PD-related diseases such as gingivitis, diabetes mellitus, and cardiovascular diseases.

### Validation of biomarker expression by qRT-PCR

**Fig. 8:**
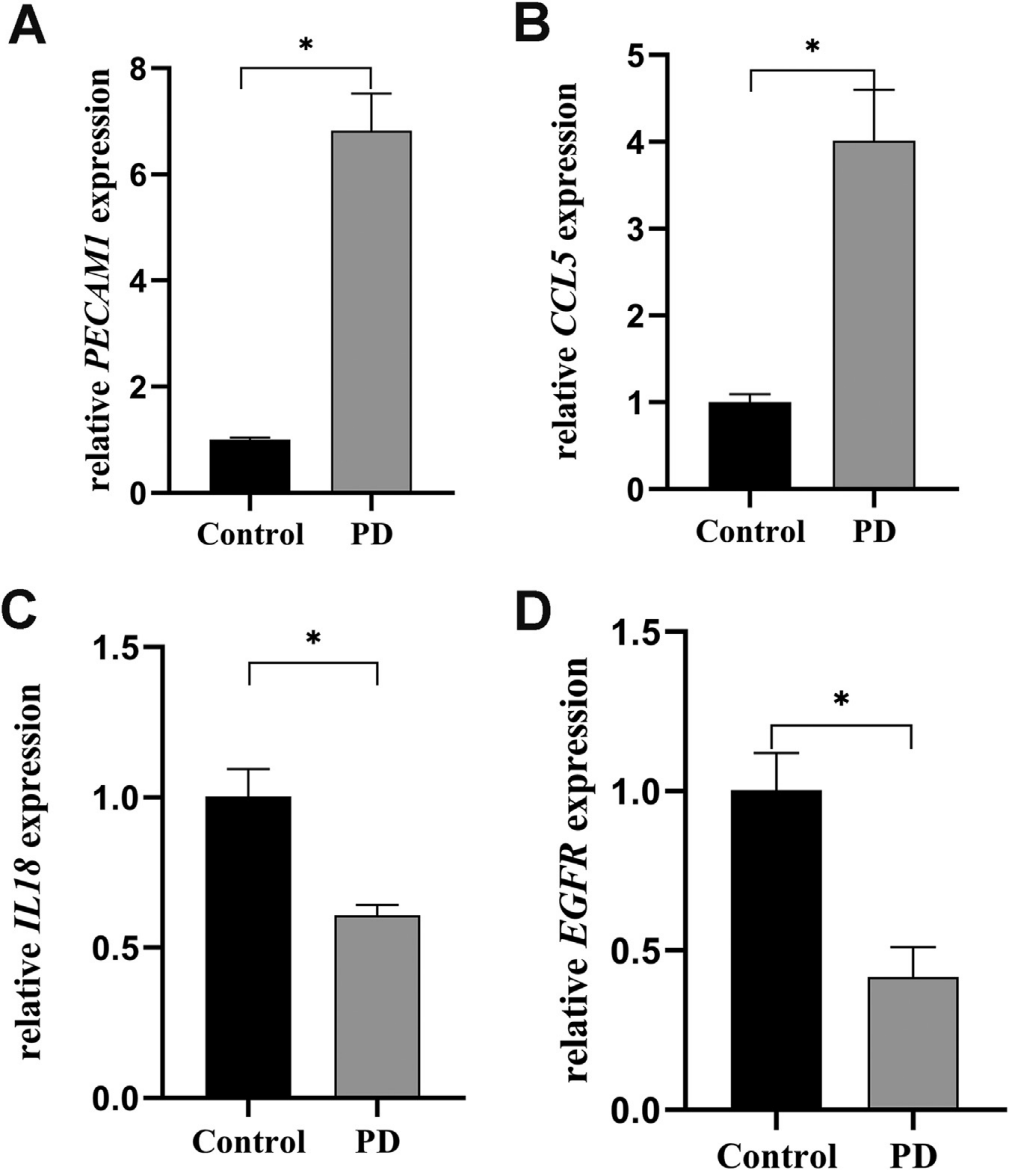
The results of qRT-PCR validation. (A-D) The relative expression levels of *PECAM1, IL18, EGFR*, and *CCL5* between different groups using qRT-PCR.

To further substantiate the validity of our findings, qRT-PCR was performed to evaluate the expression profiles of selected biomarkers across distinct sample groups. There were no significant differences in gender composition and age distribution between the two groups of samples in Supplementary Table 1 (*P* > .05). In addition, the PD group exhibited significantly higher levels of probing depth, bleeding on probing, and plaque index compared to the control group (*P* < .05). The mRNA levels of *PECAM1* and *CCL5* were observed to be significantly upregulated in the PD group compared to the control group (*P* < .05, Figure 8A and B). In contrast, the expression levels of *IL18* and *EGFR* in the PD group were markedly downregulated relative to the control group (*P* < .05, Figure 8C and D).

## Discussion

The cellular stress response serves as a pivotal mediator linking periodontal pathogenic bacterial infections to the subsequent degradation of periodontal tissues.^32^^,^^33^ SGs formation can be triggered by a range of factors acting through diverse mechanisms.^34^^,^^35^ Currently, the precise function of SGs in regulating inflammatory responses has not been fully elucidated. In different pathological conditions, SGs or their core components may demonstrate either anti-inflammatory or proinflammatory activities.^36^^,^^37^ This study, based on the transcriptome data from the GEO database and SGs-RGs, utilised multidimensional methods including differential expression analysis, machine learning, GSEA analysis, and immune infiltration analysis to identify biomarkers related to SGs in PD, and to explore their roles in the onset and progression of the disease, offering novel insights into disease diagnosis and therapeutic strategies.

Four core biomarkers (*PECAM1, IL18, EGFR*, and *CCL5*) were identified through rigorous screening. The AUC and calibration curve of the nomogram model preliminarily indicated that the model had good predictive performance. PECAM1 is prominently expressed on the surface of platelets, monocytes, and neutrophils, and serves as a key structural component of endothelial cell junctions. It plays an essential role in facilitating the removal of senescent neutrophils from the circulatory system.^38^, ^39^, ^40^ This study found that *PECAM1* was significantly upregulated in PD patients and positively correlated with the infiltration levels of plasma cells and resting dendritic cells, which was consistent with previous findings.^31^^,^^41^ PECAM1 has an important role in the inflammatory microenvironment by regulating angiogenesis and immune cell chemotaxis.^42^^,^^43^ Its high expression in PD may reflect the active state of vascular remodelling and immune cell recruitment during the disease process. Moreover, previous studies have shown that PECAM1 has a pro-inflammatory effect in rheumatoid arthritis and inflammatory bowel disease, and inhibiting its activity can alleviate the inflammatory response, suggesting that PECAM1 may be a potential target for PD treatment. However, the specific regulatory mechanism of PECAM1 in PD still needs to be further clarified through functional experiments.

IL-18 functions as a central regulator of multiple immune cell populations. Accumulating evidence suggests that its proinflammatory activity contributes to the inflammatory responses within periodontal tissues.^44^^,^^45^ IL-18 upregulates the expression of matrix metalloproteases (MMPs), including MMP-8, MMP-9, and MMP-13, and increases RANKL expression. RANKL subsequently binds to its receptor RANK, activating intracellular signalling pathways that initiate osteoclastogenesis and lead to alveolar bone loss.^46^ Nevertheless, our findings revealed a significantly reduced expression level of *IL18* mRNA in gingival tissues from individuals in the PD group compared to those from healthy controls. This observation aligned with previous findings that decreased *IL18* mRNA levels might reflect enhanced translation into protein, thereby increasing IL-18 protein availability in periodontitis.^47^ However, this mechanism warrants further experimental confirmation in subsequent studies. In the present study, *IL18* might sustain a chronic inflammatory state by modulating the functions of memory B cells, thereby offering novel insights for immunomodulatory therapies.

Genetic ablation of *EGFR* significantly attenuates the progression of periodontitis and alveolar bone loss, which are otherwise exacerbated by its suppression of the anti-inflammatory αvβ6 integrin and promotion of periodontal inflammation.^48^^,^^49^ Conversely, EGFR expression can also be significantly reduced in periodontitis tissues, which may impair osteoblast differentiation and compromise host defence mechanisms, potentially contributing to disease progression through different pathways.^50^ Further qRT-PCR analysis confirmed that *EGFR* levels were markedly reduced in the gingival tissues of PD patients compared to healthy controls, indicating a likely key role of EGFR in the pathogenesis of periodontitis.

Research on gingival fibroblasts has demonstrated that inflammatory stimuli, particularly proinflammatory cytokines like TNF-α and IL-1β, upregulate CCL5 expression and enhance its secretion.^51^ Moreover, bacterial colonisation serves as another inducer of increased CCL5 expression and secretion in gingival cells from patients with periodontitis.^52^ One of the defining features of chemokines is their capacity to recruit immune cells that display elevated expression of specific receptors. Investigations into gingival tissues from individuals with periodontitis have revealed that these patients show significant infiltration by immune cells bearing the CCR5 receptor. In this study, *CCL5* was significantly positively correlated with naive B cells, plasma cells, and memory CD4 T cells, suggesting that *CCL5* might serve as a new target for immunomodulatory therapy.

GSEA analysis revealed that the four biomarkers were predominantly enriched in translation-related pathways, including ribosomal biogenesis, translation initiation, and rRNA maturation, which were highly consistent with the core function of SGs as mRNA-protein complexes. Phosphorylation of eIF2α serves as the primary trigger for SGs formation by inhibiting translation initiation and promoting the assembly of stalled translation complexes. Four serine/threonine kinases, including heme-regulated eIF2α kinase (HRI), general control nonderepressible 2 (GCN2), protein kinase R (PKR), and PKR-like endoplasmic reticulum kinase (PERK), respond to distinct cellular stressors by phosphorylating the serine residue at position 51 of eIF2α. GCN2 is primarily activated in response to intracellular stress triggered by amino acid deficiency.^53^ PERK becomes active under conditions of endoplasmic reticulum stress (ERS).^54^ PKR functions as part of the interferon-mediated immune response and is stimulated by oxidative stress-induced double-stranded RNA (dsRNA).^55^ HRI can be triggered by oxidative stress.^56^ It is reported that certain proinflammatory cytokines, including IFN-γ and TNF-α, induce SGs assembly by triggering eIF2α phosphorylation.^57^ ERS plays a central role in regulating inflammatory reactions and may directly contribute to alveolar bone loss in periodontitis and promote polarisation of surrounding macrophages towards an M1 phenotype.^58^ Thus, we speculated that components of the ERS signalling pathway could represent promising candidates for SGs-mediated anti-inflammatory intervention. Additionally, increasing evidence suggests that periodontitis may contribute to the development of oxidative stress in both local tissues and throughout the systemic circulation.^59^ In summary, we hypothesise that eIF2α-associated signalling pathways may play a regulatory role in the immunological mechanisms underlying periodontitis. The ribosome is a multi-unit complex that translates mRNA into protein. Ribosome biogenesis is the process that generates ribosomes and plays an essential role in regulating cellular homeostasis, which may be critically involved in modulating osteoblast function.^60^ Multiple signal transduction events have been reported to control ribosome biogenesis and protein synthesis, such as Wnt/c-Myc, Mdm2-p53, and PI3K-Akt-mTOR pathways.^61^, ^62^, ^63^ It has been reported that METTL3 mediates the Wnt/β-catenin/c-Myc axis to promote osteoblast ATP production, ribosome biogenesis, and osteoblast differentiation in lipopolysaccharide-stimulated conditions.^64^ Furthermore, brain-derived neurotrophic factor may contribute to the initiation and progression of periodontitis through the regulation of the mTOR signalling pathway, which is a well-studied pathway known to control ribosome biogenesis.^65^ The significant enrichment of the ribosome pathway observed in this study provided further evidence supporting the mechanism through which SGs modulate inflammatory responses in periodontitis by regulating ribosome biogenesis.

Alterations in immune cell profiles appear to play a critical role in the association between PD and SGs. Emerging meta-analytic findings indicate that changes in localised immune responses linked to periodontitis may modulate systemic immune activity, thereby influencing the onset or development of other comorbid conditions.^66^ Li et al^67^ reported that ferritinophagy-related biomarkers were significantly correlated with immune cells like resting dendritic cells and follicular helper T cells, and they might jointly modulate immune cell function and activity via metabolic pathways, oxidative stress responses, and the regulation of cholesterol homeostasis. In this study, immune infiltration analysis identified distinct profiles of 14 differentially infiltrating immune cell types in patients with PD, including plasma cells, memory B cells, and dendritic cells. B cells regulate alveolar bone homeostasis in a murine periodontitis model through pathways that do not rely on antibody secretion but are mediated by RANKL.^68^ Blocking the activity of RANKL, B-cell-activating factor, and a proliferation-inducing ligand has been found to reduce alveolar bone loss in experimental periodontitis. These findings indicate that gingival memory B cells contribute to osteoclastogenesis, with this pro-osteoclastogenic potential increasing as the disease advances.^69^ Dendritic cells function as essential antigen-presenting cells actively engaged in immune surveillance. It has been reported that epigenetic alterations induced by ERS can have persistent effects on immune cell differentiation and functional regulation, potentially influencing dendritic cell maturation. Nevertheless, research on ERS in the pathogenesis of periodontitis remains limited.^70^ Evidence from human and mouse studies indicates that myeloid dendritic cells become activated in response to periodontal inflammatory processes.^71^ *Porphyromonas gingivalis* exploits the deficiency of potent bactericidal enzymes and ROS in dendritic cells to invade these cells via specific fimbriae, and persists intracellularly by disrupting the autophagic clearance mechanism. Furthermore, due to the high migratory capacity of dendritic cells, *Porphyromonas gingivalis* can be disseminated to distant anatomical sites, such as atherosclerotic plaques, thereby initiating systemic inflammatory responses.^72^ Precise and dynamic modulation of dendritic cell activity may facilitate the fine-tuning of immune responses, thereby establishing an optimal balance between effector and tolerogenic pathways. Such an immunoregulatory equilibrium is essential for alleviating the chronic inflammatory burden in periodontitis, a therapeutic challenge that remains inadequately addressed by existing immunotherapeutic strategies. This immunomodulatory approach may be synergistically combined with bone regeneration techniques, enabling simultaneous suppression or reversal of pathological inflammation and promotion of tissue repair. This study identified several TFs among the significantly differentially expressed biomarkers, such as CEBPB, ELF1, FLI1, STAT3, and GATA1. As a core component and crucial regulatory switch of SGs, Ras-GTPase-activating protein-binding protein 1 exerts diverse biological functions by influencing cell proliferation, differentiation, apoptosis, and RNA metabolism, and serves as a key regulator in signalling pathways such as STAT3.^73^ Studies have shown that increased STAT3 activation in PD drives inflammatory bone loss.^74^ Overall, TFs may collectively regulate the biological effects of SGs through gene regulatory networks, playing a pivotal role in the pathogenesis and progression of periodontal disease. All these biomarkers were associated with PD-related diseases such as gingivitis, diabetes mellitus, and cardiovascular diseases. Preliminary analysis indicated that 17 drugs exhibited promising therapeutic potential or substantial promise for further development. Among them, the plant-derived anti-inflammatory compound resveratrol is found to mitigate ferroptosis of alveolar osteocytes caused by diabetic periodontitis via regulation of SLC7A11/GPX4, while also reducing the expression of inflammatory mediators and attenuating the overall pathogenic effects of the disease.^75^ The predicted drug targets offer a theoretical foundation for the development of SGs-targeted medications. Notably, modulating the dynamic equilibrium of SGs can emerge as a novel strategy for intervening in periodontitis.

To sum up, this study successfully identified four key biomarkers (*PECAM1, IL18, EGFR*, and *CCL5*) that linked SGs biology to the pathogenesis of PD, established a preliminary predictive model with an AUC of 0.915. Several complementary analytical layers converged on the link between SGs biology and PD. First, intersecting DEGs with curated SGs-related genes yielded a set of candidates enriched in pathways related to translation, ribosome biogenesis and oxidative stress, all of which were closely intertwined with SGs dynamics. Second, GSEA of the four biomarkers further highlighted translation-related signatures, supporting their functional connection to SGs assembly. Third, the immune infiltration analysis demonstrated that these biomarkers correlated with different immune cell types, such as specific B cells and dendritic cell subsets, suggesting that SGs-related signalling might modulate both local inflammatory responses and systemic immune profiles in PD. Finally, TFs and drug-target network analyses speculated upstream regulators and potentially druggable interactions that were consistent with a role for cellular stress responses in periodontal inflammation. Nevertheless, several limitations should be acknowledged. The constrained availability of data from the GEO database can exacerbate individual-level variations and introduce potential biases. Furthermore, the in-house validation cohort was relatively small (*n* = 6 per group), which may limit the generalizability of the qRT-PCR findings. Importantly, protein-level confirmation using techniques such as Western blotting or immunohistochemistry, as well as functional studies (eg, gain- or loss-of-function experiments), were not performed; these represent essential future directions to establish the biological significance of the identified biomarkers. Additionally, the diagnostic nomogram, while demonstrating promising accuracy (AUC = 0.915), requires external validation in larger, independent patient cohorts before clinical application.

## Conclusion

In summary, through bioinformatics analysis combined with qRT-PCR validation, we identified four biomarkers (*PECAM1, IL18, EGFR*, and *CCL5*) and signalling pathways shared between PD and SGs. Immune infiltration analysis identified 14 differentially infiltrated immune cell types. Notably, *IL18* and *EGFR* were found to be associated with memory B cells, while *PECAM1* and *CCL5* exhibited a significant correlation with plasma cells. Additionally, TFs were screened, and the drug-gene network has been preliminarily characterised. This work provides novel hypotheses and a strong foundation for understanding the interplay between SGs and periodontal immunopathology. The constructed regulatory networks and drug predictions offer valuable starting points for future research. Ultimately, proteomic validation and functional experiments are essential prerequisites to confirm the biological and clinical significance of these candidates before any therapeutic application can be considered, which represents a critical next step in this research trajectory.

## Consent to participate

Informed consent was obtained from all individual participants included in the study.

## Author contributions

*Writing – original draft, data curation, conceptualisation*: Wang; *Methodology, data curation*: Yang and Shi; *Formal analysis, data curation*: Zhu; *Software, methodology, investigation, supervision*: Zhuang; *Writing – review and editing, supervision, and conceptualisation*: Chen.

## Ethics statement

The study was sanctioned by the Ethical Committee of the First Affiliated Hospital of Nanjing Medical University (2024-NT-09) and complied with the 1964 Declaration of Helsinki and subsequent amendments and ethics standards.

## Funding

This research did not receive any specific grant from funding agencies in the public, commercial, or not-for-profit sectors.

## Conflict of interest

The authors declare that they have no known competing financial interests or personal relationships that could have appeared to influence the work reported in this paper.

## Appendix Supplementary materials

Image, application 1

Image, application 2

Image, application 3

Image, application 4

Image, application 5

## Footnotes

Supplementary material associated with this article can be found in the online version at doi:10.1016/j.identj.2025.109390.
