# Supplementary material for: Exploration Novel Therapeutic Targets for Periodontitis via Stress Granules Biomarkers
Source: Int Dent J. 2026 Jan 22;76(2):109390. doi: 10.1016/j.identj.2025.109390 (PMC12861231; doi:10.1016/j.identj.2025.109390)
Supplement: Supplementary file 2 [file mmc2.docx]

**Supplementary table** **1-Demographic and clinical characteristics of participants.**

| **Characteristics** | **Control(n=6)** | **PD(n=6)** |
| --- | --- | --- |
| Age(years) | 30.67±2.80  (28-36) | 35±4.52  (30-40) |
| Gender (% male) | 50% | 50% |
| PD (mm) | 1.20±0.18^*^  (1.01-1.49) | 2.72±0.30 ^*^  (2.30-3.04) |
| CAL (mm) | - | 3.07±0.28  (2.64-3.37) |
| PLI | 0.76±0.22^*^  (0.48-1.08) | 1.31±0.40^*^  (0.66-1.80) |
| BOP% | 14.09±7.07^*^  (5.36-23.81) | 40.18±9.51^*^  (23.21-48.81) |

^*^ Significant difference exists between the control group and PD group. **P* < 0.05
